# Determination of Antioxidant 264 in the Butyl Rubber Stopper and the Compatibility with Recombinant Potent Antitumor and Antivirus Protein Injection

**DOI:** 10.1155/2020/8827925

**Published:** 2020-09-09

**Authors:** Min Zhao, Xianhui Li, Di Zhang, Longshan Zhao

**Affiliations:** ^1^School of Pharmacy, Shenyang Pharmaceutical University, Shenyang 110016, China; ^2^Shenyang Wellwolf Pharmaceutical Science and Technology Co. Ltd., Shenyang 110022, China

## Abstract

**Objective:**

To establish a method for extraction and determination of antioxidant 264 (2,6-di-tert-butyl-4-methylphenol) in the brominated butyl rubber stopper for injection and migration study in recombinant potent antitumor and antivirus protein injection (Novaferon).

**Methods:**

Dichloromethane-ethanol was adopted as the extraction solvent during the process of reflux extraction of antioxidant 264 in the brominated butyl rubber stopper. High-performance liquid chromatography (HPLC) was used for the determination of the migration of antioxidant 264 to Novaferon. The mobile phase consisted of acetonitrile-water (80 : 20, v/v). The flow rate was 1.5 mL/min. The detection wavelength was 280 nm.

**Results:**

The linearity range was from 4.003 to 200.150 *μ*g/mL (*r*^2^ = 0.99996), and the average recovery of antioxidant 264 was 97.8%. The applicability of the methodology was good, which can be used for the determination of antioxidant 264. The results indicated that antioxidant 264 was not detected in Novaferon after the accelerated test and three months of long-term test.

**Conclusion:**

The established validated method in this study can be used for the determination of antioxidant 264 in the rubber stopper, and the brominated butyl rubber stopper has good compatibility with Novaferon.

## 1. Introduction

The efficacy and quality of drugs are closely related to human health and safety. The substance which consisted of internal medicine and packaging material of the direct contact drug is called medicine [1, 2]. Packaging materials have been crucial components for drug products. Drug manufacturers use antioxidants, plasticizers, accelerators, and activators in order to achieve the required performance of their products. Consequently, the quality of the packaging material might have various negative effects on the pharmaceutical products and also be harmful to the human body [3, 4]. The package of pharmaceutical products should be suitable for the standard of clinic and should have the following characteristics: protection, compatibility, safety, and functionality. A number of literature studies have reported the compatibility of pharmaceutical packaging [5–9]. The research of compatibility should include the interaction of packaging materials and drugs [10]. Compatibility studies demonstrate that there is no serious interaction between the packaging material and drugs. It is an objective requirement for the improvement of the inspection standard [11, 12]. Therefore, the compatibility research of drugs and packaging materials should be carried out in the early stage of drug development or the selection of packaging materials throughout the entire process of drug development [13].

When selecting and confirming the packing seals, the pharmaceutical-producing enterprises should protect the quality of drugs and achieve the purpose of drug delivery in the process of packaging, storage, and transportation. Rubber is a kind of linear flexible macromolecule polymer. Its molecular chain is flexible and can produce large deformation under the external force. It can quickly restore its original shape when external force disappears [7]. Rubber is characterized by its excellent elasticity over a wide temperature range. As a packaging component, on the one hand, the rubber stopper should satisfy the sealing requirements of the packaging system; on the other hand, it should also have good compatibility with the drug, and it cannot be introduced into the safety risk of the extract, or the level of extract meets the safety requirements; the quality, efficacy, and safety of the drug will not be influenced by adsorption of active ingredients in pharmaceuticals or functional accessories [14–16]. Pharmaceutical manufacturers should assess potential safety risks and make corresponding compatibility studies based on the dosage form of the drug and the degree of risk of the route of administration. Compatibility test of the drug-packaging material and drugs refers to an experiment to investigate whether there is transfer or adsorption between drug-packaging materials and drugs, which can affect the quality of drugs [17, 18]. The compatibility research of pharmaceuticals and packaging materials mainly includes three aspects: extraction study, interaction study (including migration test and adsorption test), and safety assessment [19].

Antioxidant 264 (2,6-di-tert-butyl-4-methylphenol) is an excellent generic phenolic antioxidant, which is most widely used as an antioxidant in plastics. It has the advantages of good antioxidant effect, high thermal stability, good compatibility with plastics, nonpollution to plastics, and so on [20, 21]. The European Pharmacopoeia also regulates that the maximum amount of the antioxidant in pharmaceutical packaging is 0.3% [22]. Meanwhile, antioxidant 264 has the effect of inhibiting the activity of the human respiratory enzyme and increasing the activity of liver microsomes [23, 24]. The FDA Cancer Evaluation Committee is also considering the carcinogenicity of antioxidant 264 [25]. As rubber stoppers have the advantages of simple production process, abundant raw material sources, and high rate of quantity and price [26], Wang et al. identified that it is very important to determine the content of antioxidant 264 in rubber stoppers. Pu et al. established a method to determine antioxidant 1010 and antioxidant 5057, which provided a reference for the detection of antioxidant migration [27]. Novaferon is based on 12 kinds of the IFN*α* gene (interferon alpha gene), which is a way to use DNA reorganization technology development of genetic engineering drugs [28], and the compatibility research between antioxidant 264 in rubber stoppers and Novaferon can better guarantee the safety, efficacy, and stability of the drug.

Based on the necessity of the aforementioned control of antioxidant 264, this study established a reflux extraction method of organic solvents to extract antioxidant 264 and determine it by HPLC. The method was simple and accurate for the determination of antioxidant 264 in brominated butyl rubber stoppers. Then, the developed method was successfully applied to the compatibility between brominated butyl rubber stopper and Novaferon to evaluate the safety of migration of antioxidant 264 [29, 30].

## 2. Experimental

### 2.1. Materials and Reagents

Antioxidant 264 (99% purity) was obtained from Sigma Company (St. Louis, MO, USA). The batch number of the brominated butyl rubber stopper for injection was 16111006. Novaferon samples (lot numbers: 201708001, 201708002, 201708003, 201708004, 201708005, and 201708006) were provided from Jiehua Biotechnology Group Co., Ltd. (Qingdao, China).

Chromatographic-grade ethanol and dichloromethane were obtained from Dikma Technologies (Beijing, China). Acetonitrile was purchased from ANPEL Laboratory Technologies (Shanghai, China). Deionized water was purchased by China Resources C'estbon Beverage Co., Ltd. (Shenzhen, China).

### 2.2. Instrumentation

Liquid chromatographic analysis was performed using the Agilent Series HPLC system (Agilent, San Jose, CA, USA) equipped with a diode array detector (DAD). Separations were carried out on an Ultimate XB-C18 (150 mm × 4.6 mm, 5 *μ*m; Welch, China) analytical column, which was maintained at 35°C. And the UV detector wavelength of antioxidant 264 was set at 280 nm. Solid phase extraction column and octadecylsilane chemically bonded silica of stuffing were also obtained from Welch. The mobile phase consisted of acetonitrile (A) and H_2_O (B) (80 : 20, v/v). The flow rate and injection volume were set at 1.5 mL/min and 20 *μ*L, respectively. Meanwhile, the condition of flow rate was isometric elution. Liquid chromatographic analysis was used for compatibility experiments.

### 2.3. Preparation of the Solution

#### 2.3.1. Preparation of the Reference Solution

Accurately weighed amount of antioxidant 264 was taken and placed in a 100 mL volumetric flask; then, the standard stock solution of antioxidant 264 was prepared with anhydrous ethanol at the concentration of 0.4 mg/mL. An amount of the antioxidant 264 standard stork solution to a series of reference substance dilutions contained S1, S2, S3, S4, S5, S6, and S7 and then were diluted by anhydrous ethanol to the antioxiant 264 concentration of 0.004, 0.012, 0.04, 0.08, 0.12, and 0.20 mg/ml. Serial dilutions of the standard stock solution of antioxidant 264 were used for subsequent experimental.

#### 2.3.2. Preparation of the Test Solution

*(1)*. *Preparation of Samples of the Brominated Butyl Rubber Stopper.* The medicinal rubber stopper was shredded, accurately weighed about 2.5 g in a 100 mL mill borosilicate glass flask. Subsequently, 10 mL dichloromethane-ethanol (1 : 1) was added accurately, then boiled and refluxed for 4 h, and then cooled down to room temperature with dichloromethane-absolute ethanol (1 : 1) added to reduce the amount of loss. Finally, the solution of the brominated butyl rubber stopper was passed through the 0.45 *μ*m microporous membrane filter and was used as a test sample.

*(2)*. *Novaferon Sample for the Test Solution*. Novaferon was filled into the brominated butyl rubber stopper which was used as the sample stopper. Then, 1 mL Novaferon was extracted by the solid-phase extraction column which was activated with 4 mL methanol and 4 mL water, respectively. Furthermore, Novaferon was eluted with anhydrous ethanol solution, and the volume was fixed to 5 mL by anhydrous ethanol solution. Finally, the sample was passed through a 0.22 *μ*m membrane filter prior to injection. Novaferon sample was prepared mainly for migration studies.

## 3. Results and Discussion

### 3.1. Method Validation

The validation study consisted of selectivity, limit of detection (LOD), limit of quantitation (LOQ), precision, repeatability, and recovery, as well as stability.

#### 3.1.1. Specificity

**Figure 1:**
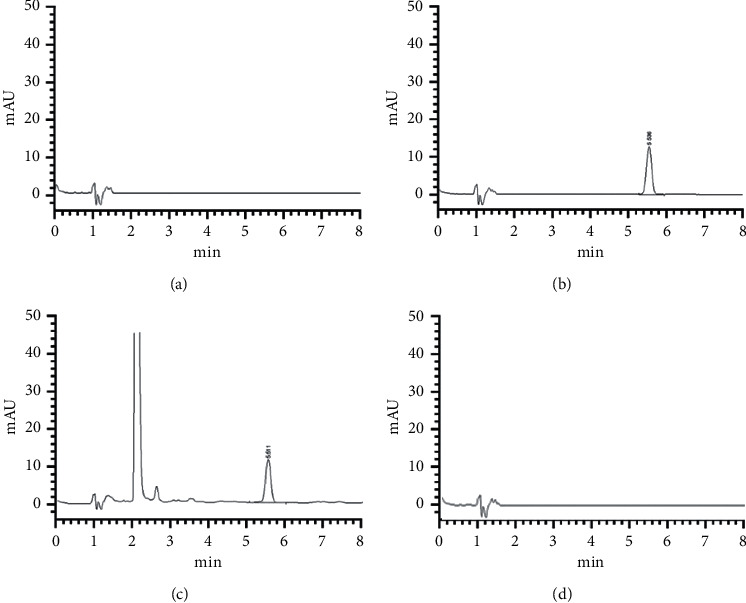
HPLC chromatogram of the blank solvent (a), reference standard solution of antioxidant 264 (b), sample solution of the rubber stopper (c), and recombinant potent antitumor and antivirus protein injection for the test (d).

The specificity of the method was proved by comparing typical liquid chromatograms of the blank solution and reference standard solution of antioxidant 264, as well as sample solutions of rubber stoppers and Novaferon. The results showed that there was no interference in the detection of antioxidant 264 in the blank solution, rubber stopper test solution, and Novaferon test solution, and the method presented good specificity and resolution. The chromatograms are shown in Figure 1.

#### 3.1.2. Linearity and Range

The linear solution was injected into the liquid chromatograph with 20 *μ*L according to the method described above in Section 2.3.1, and the peak area was determined. The linear regression analysis was carried out with the antioxidant 264 mass concentration (*x*) as the horizontal coordinate and the peak area (*y*) as the vertical coordinate. The equation of the calibration curves was as follows: *y* = 3.23*x* + 0.9316 (*r*^2^ = 0.99996), indicating that antioxidant 264 had a good linear relationship with the peak area in the concentration range of 4.003–200.150 *μ*g/mL.

#### 3.1.3. LOD and LOQ

The LOD and LOQ were determined at a signal-to-noise ratio of 3 : 1 and 10 : 1 by injecting a series of diluted standard solutions with known concentrations, respectively. When the response values were about 3 and 10-fold signal-to-noise ratio, the LOD of antioxidant 264 was 192.2 ng/mL, and the LOQ was 434.0 ng/mL.

#### 3.1.4. Instrument Precision

The precision of the method was obtained by injecting the same reference solution of antioxidant 264 for six times continuously. The results showed that the RSD of the antioxidant 264 peak area was 0.07%, indicating high instrument precision.

#### 3.1.5. Repeatability

Six test solutions of plastic stopper and Novaferon were prepared in parallel. Under the above chromatographic conditions, 20 *μ*L of the above solutions was accurately absorbed and injected into the liquid chromatograph, respectively. Then, chromatogram was recorded. The content and RSD of the peak area for antioxidant 264 in six replicates of the rubber stopper sample were 149.76 *μ*g/mL and 0.55%, respectively. As a result, antioxidant 264 was not detected in Novaferon, implying the method has good repeatability.

#### 3.1.6. Recovery

Accurately weighed 2.5 g medicinal rubber stopper and was cut into small pieces in a 100 mL mill borosilicate glass flask, parallel operation 3 times, and then added the antioxidant 264 reference substances 0.8 mL, 1 mL, and 1.2 mL, respectively, and then added 10 mL methyl chloride-ethanol (1 : 1) of each sample. Mixture was boiled under a reflux condenser for 4 h and cooled down to room temperature, supplemented with methylene chloride-ethanol (1 : 1). Finally, the solution was filtered by the 0.45 *μ*m microporous membrane. Each concentration was prepared in 3 copies in parallel.

Three parts of 2 mL Novaferon, which was filled into the rubber stopper, were added, respectively. Then, the antioxidant 264 standard stock solution (0.8 mL, 1 mL, and 1.2 mL) was extracted by the solid-phase extraction column which was activated with 4 mL methanol and 4 mL water, respectively. Then, Novaferon was eluted with anhydrous ethanol solution, and the volume was fixed to 10 mL by anhydrous ethanol solution. Finally, the solution was filtered by the 0.22 *μ*m microporous membrane. Each concentration of the sample was prepared in triplicate.

The sample-adding recoveries for antioxidant 264 in samples are shown in Tables 1 and 2. As can be seen from the tables, the average recoveries of antioxidant 264 in the rubber stopper and Novaferon were 97.82% and 102.19%, respectively. In this section, the regulations of the spike recovery limit for chemical drugs in the 2015 edition of the Pharmacopoeia of the People's Republic of China were referenced, which stated that when the concentration of the analyte was between 1 *μ*g/g and 10 *μ*g/g, 75%–120% of the recovery was acceptable [31], implying that our analytical method shows high accuracy.

#### 3.1.7. Stability

The reference solution (S4) and the sample solutions of Novaferon and the rubber stopper were used for testing the stability of this method, which were placed for 0, 10, and 22 h at room temperature, and the results showed that the RSD was 1.86% for antioxidant 264 in the rubber stopper, and it was not detected in the injection, which indicated that both the test solutions were stable in 22 h.

### 3.2. Content Determination

#### 3.2.1. Extraction of Test Samples

The method of preparation of the rubber stopper for the test solution is the same as the established detection method, and 20 *μ*L aliquot of the solution was subjected to the HPLC system for analysis. The result showed that antioxidant 264 was detected in the sample solution of the rubber stopper. The content of antioxidant 264 in the rubber stopper was 0.014%, which met the limit requirement of antioxidant 264 in the European Pharmacopoeia (not more than 0.125%).

#### 3.2.2. Migration Test

Migration test was used to detect the migration of antioxidant 264 in the rubber stopper into Novaferon. In this experiment, the migration of antioxidant 264 from the rubber stopper into the product was investigated by the accelerated test (25°C ± 2°C, RH 60% ± 10%) for 0, 1, 2, and 3 months and long-term stability (2°C–8°C) for 0 and 3 months.

According to the established detection method, Novaferon was prepared at each time point, and 20 *μ*L of Novaferon was absorbed and injected into the liquid chromatograph, respectively. The results showed that antioxidant 264 was not detected in six-batch samples after the accelerated test and three months of the long-term test.

According to the accelerated test and the long-term stability test, the content of antioxidant 264 was not detected in the six-batch samples of Novaferon after storage for a period of time under certain conditions. From a safety point of view, the potential migrant in the injection could be predicted, and the amount of antioxidant 264 was 192.2 ng/mL on the basis of the limit of detection.

### 3.3. Migration Assessment

According to the European Chemicals Agency (ECHA) reports of the International Uniform Chemical Information Database (IUCLID), the daily oral intake of antioxidant 264 in humans was 0–0.3 mg/kg/day. The equation of permitted daily exposure (PDE) for humans is as follows [32–34]:

(1) $$\begin{array}{c} \text{NOEL}=\frac{{\text{LD}}_{50}*70}{2000}, \end{array}$$

(2) $$\begin{array}{c} \text{PDE}=\frac{\text{NOEL}*W}{F1*F2*F3*F4*F5*F}. \end{array}$$

The median lethal dose (LD_50_) value is 6000 mg/kg/BW (rat, oral, 24 hr) for 6000 mg/kg/BW antioxidant 264 [35]. The NOEL represents no-observed-effect level, where *W* represents an arbitrary adult weight of 50 kg (regardless of sex), *F*1 represents a factor for extrapolation between species, and *F*1 equals 5, meaning extrapolation from rats to humans; *F*2 has a factor of 10 to account for variability between individuals; *F*3 equals 10, applying to account for toxicity studies of short-term exposure; *F*4 represents a factor that may be applied in cases of severe toxicity, and *F*4 equals 1, meaning serious toxicity is not found to this day; and *F*5 is a factor that may be applied if the no-effect level is not established (*F*5 = 1), and the composite factor (*F*) between oral and injection is 100 (*F* = 100). Therefore, the PDE values of antioxidant 264 are calculated as follows:

(3) $$\begin{array}{c} \text{NOEL}=\frac{6000*70}{2000}=210\;\text{mg}/\text{kg}/\text{day}, \end{array}$$

(4) $$\begin{array}{c} \text{PDE}=\frac{210*50}{5*10*10*1*1*100}=0.21\text{ mg}/\text{day}. \end{array}$$

The specification of Novaferon was 20 *μ*g/1.0 mL/bottle and 10 *μ*g/1.0 mL/bottle of Novaferon, and the dosage of the intramuscular injection was one time/day, 10 *μ*g/time. The maximum amount of 20 *μ*g/1.0 mL/bottle of Novaferon was 0.5 mL, and the maximum amount of 10 µg/1.0 mL/bottle of Novaferon was 1 mL. As a result, antioxidant 264 was not detected. The maximum daily intake of antioxidant 264 was less than 0.2 *μ*g. The calculation results showed that the daily intake of antioxidant 264 was lower than the PDE value, and the migration of antioxidant 264 was considered without the safety risk.

## 4. Conclusion

Brominated butyl rubber stopper can be widely used in primary packaging of various drugs. Due to the compatibility issues that may arose in the process of direct contact with drugs, compatibility studies are necessary to ensure the safety, stability, and efficacy of drugs. In this experiment, the content of antioxidant 264 and its migration in Novaferon were determined by HPLC. The method had high precision, good repeatability, high accuracy, and wide applicability.

This method was used to detect one batch of the rubber stopper and six batches of Novaferon. The results showed that the above used brominated butyl rubber stopper contained antioxidant 264, but its content was within the limit of the European Pharmacopoeia. Antioxidant 264 was not detected in six-batch samples, which were stored under accelerated (0, 1, 2, and 3 months) and long-term conditions (0 and 3 months); meanwhile, the daily intake was lower than the PDE value, indicating that the compatibility between Novaferon and the rubber stopper was good.

The method successfully demonstrated that rubber stoppers used as a drug-packaging material can be assessed during injectable product stability studies, thereby ensuring the safety of the drug.

## Figures and Tables

**Table 1:** Recovery of antioxidant 264 from the rubber stopper.

Sample	*m* sample/*μ*g	*m* added/*μ*g	*m* found/*μ*g	Recovery (%)	Average recovery (%)	RSD (%)
Antioxidant 264 (1)	320.24	354.79	670.27	98.51	97.82	1.56
	320.40	366.11	670.27	94.93		
	320.16	360.66	670.88	96.89		
Antioxidant 264 (2)	400.30	346.54	744.07	99.31		
	400.50	355.80	744.07	96.95		
	400.20	349.91	739.82	97.43		
Antioxidant 264 (3)	480.36	364.79	838.82	98.68		
	480.60	367.99	837.31	97.65		
	480.24	360.01	840.34	100.02		

**Table 2:** Recovery of antioxidant 264 in recombinant potent antitumor and antivirus protein injection.

Sample	*m* added/*μ*g	*m* found/*μ*g	Recovery (%)	Average recovery (%)	RSD (%)
Antioxidant 264 (1)	320.24	337.11	105.27	102.19	2.23
	320.40	336.81	105.12		
	320.16	337.11	105.29		
Antioxidant 264 (2)	400.30	403.32	100.75		
	400.50	402.71	100.55		
	400.20	403.01	100.70		
Antioxidant 264 (3)	480.36	483.49	100.65		
	480.60	484.40	100.79		
	480.24	483.19	100.61		

## Data Availability

The data used to support the findings of this study are included within the article.
